# Sinorhizobium prairiense sp. nov., a nitrogen-fixing symbiont of Phaseolus vulgaris isolated from Canadian prairie soil

**DOI:** 10.1099/ijsem.0.006947

**Published:** 2025-10-30

**Authors:** Anna Motnenko, Justin P. Hawkins, Patricia A. Ordoñez, Ivan J. Oresnik

**Affiliations:** 1 Department of Microbiology, University of Manitoba, Winnipeg, MB, Canada

**Keywords:** *Rhizobium*, Sinorhizobium, *Phaseolus vulgaris*

## Abstract

Three symbiotic bacteria (K101^T^, C101 and M103) were obtained from nodule-trapping experiments using *Phaseolus vulgaris,* which was inoculated with soil samples from three distinct field sites in Manitoba, Canada. Here, we provide a phenotypic characterization and genomic analysis of these bacteria. Based on a core phylogeny (424 core genes), digital DNA–DNA hybridization and average nucleotide alignment, these isolates group within the *Sinorhizobium* clade and are closely related to *Sinorhizobium meliloti*. Each strain contains four replicons that include a chromosome (3.5 Mb), a putative chromid (1.7 Mb) and two plasmids (plasmid A, 0.56 Mb; plasmid B, 0.77 Mb). The chromosome, chromid and plasmid B are closely related to the replicons found in *S. meliloti*, as shown by phylogenies constructed from the concatenation of either the *parAB* genes for the chromosome or the *repABC* genes for the chromid and plasmid B. The remaining plasmid was found to group with a plasmid from *Sinorhizobium americanum*. Consistent with this, the nodulation genes on this plasmid were also more similar to those in *S. americanum*, as seen in a phylogeny generated from the concatenation of the *nodABC* genes. Examination of the *nodC* phylogeny suggests a close association with the mediterranensis symbiovar. All three isolates were capable of symbiotic nitrogen fixation with * P. vulgaris*. Based on genomic and phenotypic data, we propose these isolates as a novel species within the *Sinorhizobium* clade, named *Sinorhizobium prairiense* sp. nov., for which the type strain is K101^T^ (=LMG 33767^T^=DSM 118657^T^).

## Data Availability

Genomes have been deposited under BioProject PRJNA798730 and BioSamples SAMN30631408 (K101^T^=DSM 118657^T^=LMG 33767^T^), SAMN30631407 (C101=DSM 118658=LMG 33768) and SAMN30631409 (M103=DSM 118659=LMG 33769). Genome assemblies can be found as follows: K101^T^ (GCF_029201645.1), C101 (GCF_029201665.1) and M103 (GCF_029201625.1). NCBI accession numbers for the 16S rRNA sequences are as follows: K101^T^, PV061366; C101, PV061365 and M103, PV061367.

## Introduction

Symbiotic nitrogen fixation primarily occurs between certain legume plants and bacterial species known as rhizobia [1,2]. A significant agricultural example is the interaction between *Phaseolus vulgaris* (common bean) and *Rhizobium etli*. Common beans are grown globally, with an annual production of 23 million tons, making them an important food source worldwide. However, *P. vulgaris* is considered a poor nitrogen-fixing legume and is typically cultivated in suboptimal soils. This leads to yields of less than 20% of their maximum potential, indicating considerable room for improvement [3,4]. Manitoba is the second-largest producer of dry beans in Canada, accounting for 40% of the country’s production in 2020 [5].

A major issue that hinders the use of symbiotic nitrogen fixing for bean plants in Canada and the Northern USA is the lack of an effective inoculant. Because of this limitation, bean crops are often recommended to be supplemented with nitrogen fertilizer, which is costly and harmful to the environment. While many different bacteria can form a nitrogen-fixing symbiosis with *P. vulgaris* [6], *R. etli* tends to be the predominant species used in inoculants for the plant [7]. To obtain a wild bacterial strain that could serve as an inoculant strain for common bean on the Northern Plains, we isolated bacteria from the soil samples in Manitoba that could establish a symbiotic relationship with *P. vulgaris*. This work focuses on characterizing these isolated strains that represent a new, distinct species of *Sinorhizobium*.

## Isolation of rhizobia

Soil samples were collected from Ian N. Morrison’s Research Farm in Carmen, Manitoba (49.492225, –98.042452), Richardson International’s Kelburn Farm in Manitoba (49.694073, –97.122946) and an on-farm site in Melita, Manitoba (40.246706, –1010.016030) in 2017 and stored frozen at −70 °C. The collected samples were thawed and used as inoculum. Briefly, * P. vulgaris* seeds were surface-sterilized by soaking the seeds in a 1% sodium hypochlorite solution for 20 min. After decanting the hypochlorite, the seeds were thoroughly washed with 10 volumes of sterile distilled water (dH_2_O) over ~30 min. These sterile seeds were planted into sterile sand–vermiculite and inoculated with 5 g of soil resuspended in 50 ml of sterile dH_2_O. After 28 days, the plants were uprooted, selected nodules were picked, rinsed with H_2_O, surface-sterilized in 1% hypochlorite for 1 min, and then rinsed with sterile dH_2_O in a microfuge tube. The nodules were finally crushed with an inoculating stick in a volume of 200 µl. Approximately 5 µl of the suspension was placed on a Tryptone Yeast extract medium (TY) plate (5 g l^−1^ tryptone, 2.5 g l^−1^ yeast extract and 10 mM CaCl_2_) and streaked for single colonies. Each isolate was single-colony purified at least three times. Three strains, C101, K101^T^ and M103, were randomly retained as representatives from the three distinct locations for further characterization. Purified bacterial cultures were stored at −70 °C in TY broth containing 8% dimethyl sulfoxide.

## Genome sequencing and taxonomy of the isolated strains

The genomes of C101, K101^T^ and M103 were sequenced to determine the identities of the strains isolated in this study. Genomic DNA was isolated using the PureLink Genomic DNA Mini Kit (Invitrogen). All genomes were sequenced as previously described using the Nanopore MinION Mk1B [8]. Sequencing was performed on a Nanopore MinION Mk1B using the SQK-LSK-112 and EXP-NBD-112.24 kits and R10.4 flow cells, with MinKNOW (Oxford Nanopore) software. Base calling was conducted with Guppy GPU [9]. Default parameters were applied for all software used in the analysis. Sequencing was halted when, on average, enough data were available to provide ~100× coverage for all genomes. Reads were trimmed with BBDuk [10], and then *de novo* genome assembly was executed using Flye, followed by three rounds of polishing with Minimap2 [11,12]. Genome completion was estimated with CheckM [13] and was found to be above 99%, with an estimated 1% contamination in all assemblies. Completed genomes were annotated with the Prokaryotic Genome Annotation Pipeline using default parameters and automatic taxa determination [14]. The sequencing statistics are presented in Table 1.

**Table 1.** Sequencing characteristics of strains *S. prairiense* K101^T^, *S. prairiense* C101 and *S. prairiense* M103

	*S. prairiense* C101	*S. prairiense* K101^T^	*S. prairiense* M103
Coverage	210×	246×	135×
Contigs N50 (bp)	3521805	3521656	3521513
Total genome length (bp)	6553348	655113	6552870
Replicon lengths (bp)	Chromosome: 3521805	Chromosome: 3521656	Chromosome: 3521513
	Chromid: 1695852	Chromid: 1695829	Chromid: 1695772
	Plasmid B: 772700	Plasmid B: 772675	Plasmid B: 772648
	Plasmid A: 562991	Plasmid A: 562953	Plasmid A: 562937
G+C content (mol%)	62.1%	62.1%	62.1%
Protein-coding sequences	6,163	6,158	6,183
rRNA loci	3	3	3
tRNA genes	55	55	55

**Fig. 1.**
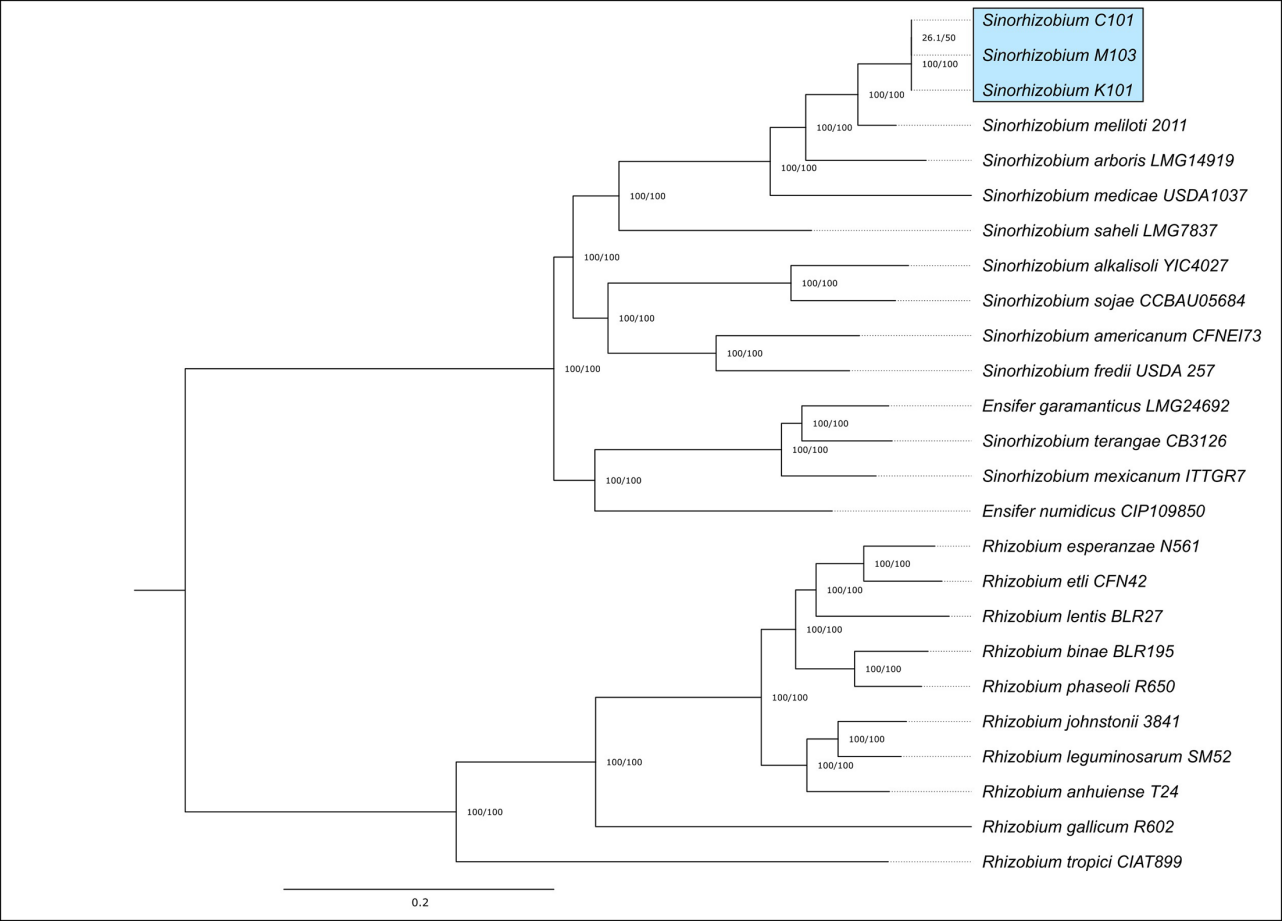
Maximum-likelihood phylogenetic tree based on the concatenation of 424 core protein-coding sequences, inferred using IQ-TREE (model GTR+F+I+G4). The species isolated in this work are highlighted in blue. Bootstrap values are indicated for each node (1,000 replicates). F, empirical base frequencies; G4, rate heterogeneity across sites using a discrete Gamma model 4 [36]; GTR, general time reversible model with unequal rates and unequal base frequencies; I, rate heterogeneity across sites allowing for a proportion of invariable sites [36].

To determine where these isolates belonged in the *Rhizobiaceae* family, phylogenies were constructed based on a core set of 424 conserved genes identified using GET_HOMOLOGUES [15] and GET_PHYLOMARKERS [16]. The results indicate that the isolates clustered closely within the *Sinorhizobium* genus and were closely related to *Sinorhizobium meliloti* Rm1021 (Fig. 1).

To assign an initial classification to the isolates, the genome sequences were submitted to the Type Strain Genome Server (TYGS) [17]. The closest type strain species was *S. meliloti* USDA 1002; however, based on computational metrics, the isolates were suggested to represent a new species. The metrics used by TYGS were digital DNA–DNA hybridization (dDDH) and average nucleotide identity (ANI). Scores for which ANI is ≤95% and dDDH is ≤70% [18] indicate a sufficient difference to consider a new species. The scores between the isolates and *S. meliloti* were 94.0% for ANI and 57.1% for dDDH, respectively, suggesting that these strains were distinct species compared with the nearest type strains (Table 2). However, the genomes of our isolates from three distinct locations in Manitoba were all highly similar and potentially represent three different strains of the same species, with ANI scores ranging from 99.96% to 99.97%.

**Table 2.** dDDH and ANI comparison with strains similar to *S. prairiense* K101^T^, *S. prairiense* C101 and *S. prairiense* M103

Strain	Comparison	dDDH (formula 2)	ANI	16S rRNA	G+C mol% difference
C101	*S. meliloti* USDA 1002^T^	57.1%	94.0%	99.9%	0.1%
	*S. americanum* CFNEL156^T^	25.8%	83.1%	98.9%	0.2%
	*S. arboris* LMG14919^T^	39.8%	89.9%	99.6%	0.1%
M103	*S. meliloti* USDA 1002^T^	57.1%	93.9%	99.9%	0.1%
	*S. americanum* CFNEL156^T^	25.8%	83.1%	98.9%	0.2%
	*S. arboris* LMG14919^T^	39.7%	89.9%	99.6%	0.1%
K101^T^	*S. meliloti* USDA 1002^T^	57.1%	94.0%	99.9%	0.1%
	*S. americanum* CFNEL156^T^	25.8%	83.1%	98.9%	0.2%
	*S. arboris* LMG14919^T^	39.8%	89.9%	99.6%	0.1%

*S. meliloti* 4H41 was initially assigned to *S. meliloti* based on a partial 16S sequence [19]. Notably, based on genome-wide ANI using the genome sequence of *S. meliloti* 4H41 [20], we found that it is 98% similar to our isolates, suggesting that it may be the same species (Fig. S1, available in the online Supplementary Material). We note that in a recent TYGS query, when the genome sequence of 4H41 was submitted along with those of C101, K101^T^ and M103, the TYGS output also classified 4H41 as an unknown species (Fig. S2). Taken together, this suggests that 4H41 should be reclassified.

## Phylogenetic groupings based on nodulation genes

Based on core gene phylogeny, our isolates form a distinct species within the *Sinorhizobium* genus but are highly similar to * S. meliloti*. Although there are reports that *S. meliloti* can nodulate *P. vulgaris*, this is uncommon [21,22]. To determine the genetic nodulation potential of these strains, all annotated nodulation and nitrogen fixation genes were identified using blastp [23] (Table S2).

The lipo-chitooligosaccharide produced by *R. etli*, which nodulates *P. vulgaris*, typically consists of 4–5 *N*-acetylglucosamine residues with *N*-methyl, o-carbamyl and acetyl-fucosylated substituents [24]. Of these, the *N*-methyl addition carried out by the methyltransferase encoded by *nodS* appears to be essential for the nodulation of *P. vulgaris* [25]. The gene encoding NodS was found within a cluster of nodulation genes within these isolates on plasmid A.

**Fig. 2.**
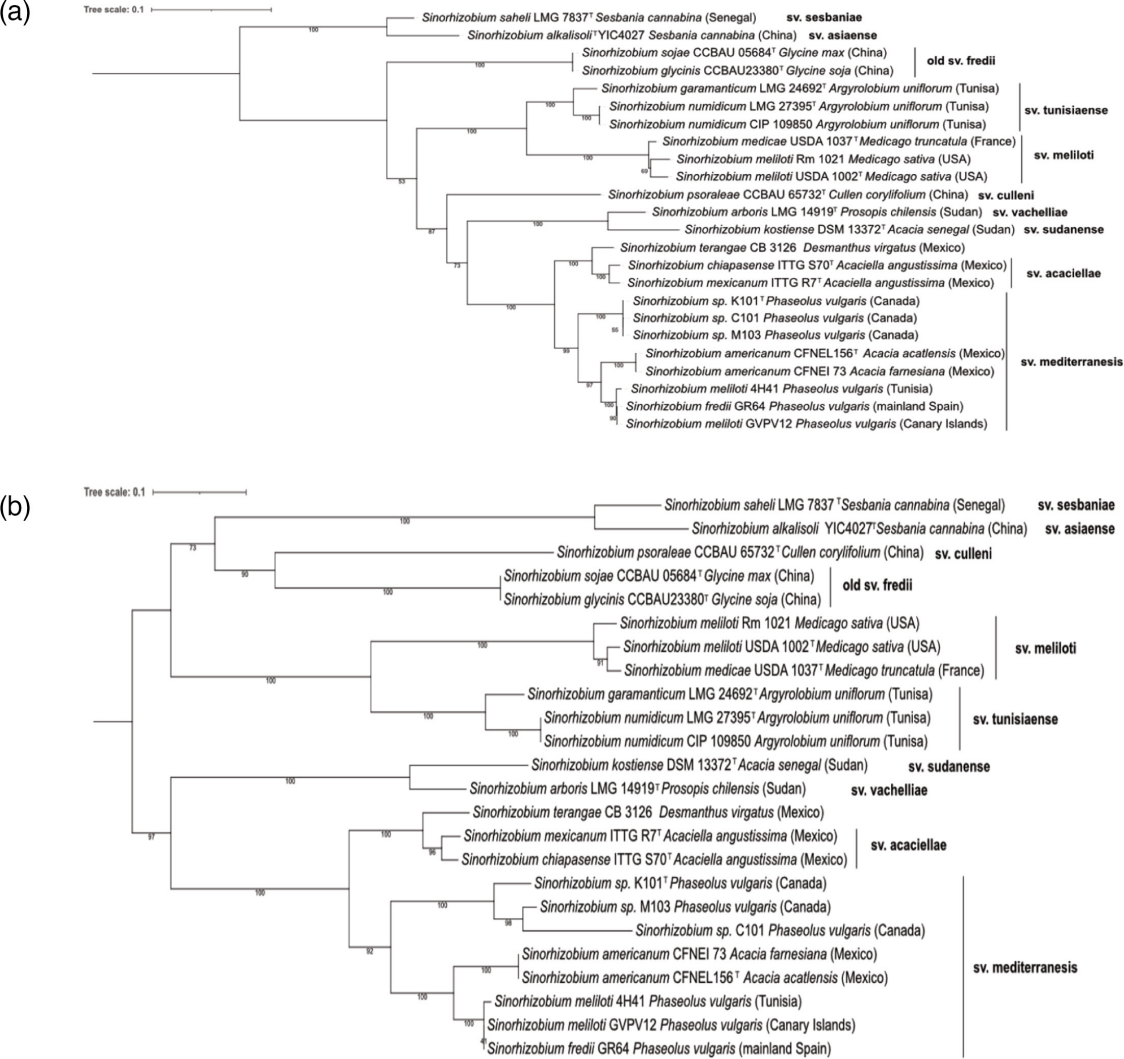
Maximum-likelihood phylogenetic tree based on (a) nucleotide sequences of *nodC* and (b) concatenation of protein sequences of NodABC. Known symbiovars are indicated in bold. Trees were generated using the IQ-TREE model with TPM3u+F+I+G4 for *nodC* and JTT+F+G4 for the concatenation of NodABC, 1,000 iterations were performed using ultrafast bootstrap, and percentage bootstrap values are given at branching points. Accession numbers used can be found in Table S2.

A custom database was developed from related *Sinorhizobium* and *Rhizobium* type strains, along with our isolates, to facilitate phylogenetic clustering based on the identified symbiotic genes. The database was queried using *nodA*, *nodB* and *nodC* from *S. meliloti* USDA1002 as input. Homologs were subsequently identified through blastp. In instances where these genes were not annotated or were misannotated, the annotation was manually curated before sequences were extracted. The *nodC* nucleotide sequences were aligned using mafft v7.526 [26]. In addition, the corresponding translated sequences of *nodA*, *nodB* and *nodC* were concatenated and aligned using mafft. These aligned sequences were then utilized to construct a phylogenetic tree with IQ-TREE Maximum Likelihood, employing the default settings for model search with 1,000 iterations (Fig. 2). The results indicate that C101, K101^T^ and M103 are associated with other rhizobia known to nodulate *P. vulgaris* and, more specifically, are most closely related to those found in *Sinorhizobium americanum*. The isolates appear to belong to symbiovar (sv.) mediterranensis.

## Genome organization

The isolates C101, K101^T^ and M103 are phylogenetically most closely related to *S. meliloti* (Fig. 1). Since C101, K101^T^ and M103 share the same overall genome architecture – each containing one main chromosome, a single putative chromid and two accessory plasmids – K101^T^ was selected as a representative for comparison with *S. meliloti*. Each replicon from strain K101^T^ was arranged to align with its putative counterpart from *S. meliloti* and analysed using FastANI [27].

**Fig. 3.**
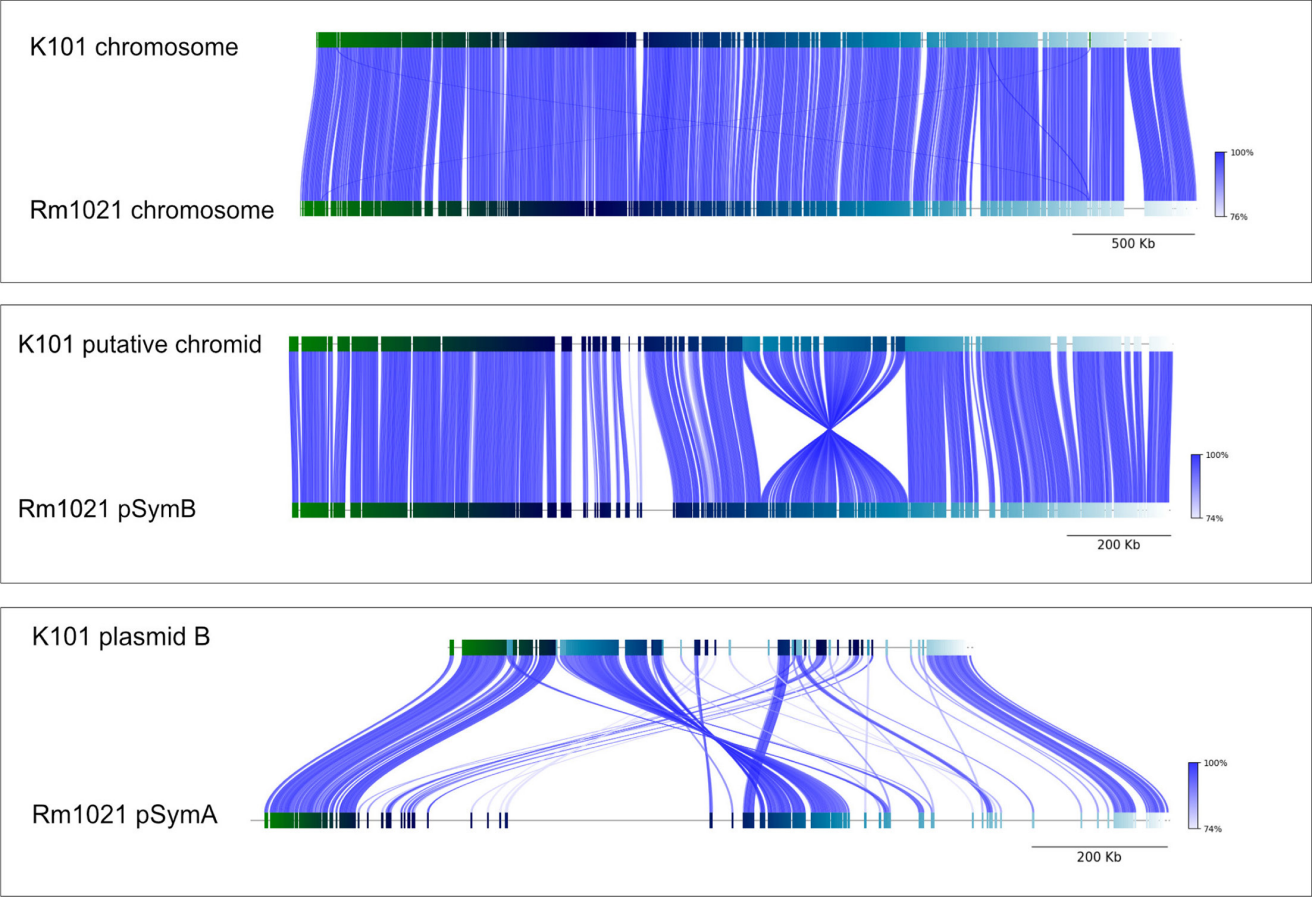
Genome architecture of *S. prairiense*. ANI alignments (made using FastANI) between replicons from *S. meliloti* Rm1021 and S. prairiense K101^T^. (**a**) Comparison of chromosomes; (**b**) comparison of the putative chromide with pSymB and (**c**) comparison of plasmid B and pSymA.

Using default settings, the analysis revealed that 92.5% (1,085 out of 1,173 sequence fragments) of the chromosome had an ANI of 95.0% (Fig. 3a). Similarly, 85.5% of the putative chromid (482 out of 565 sequence fragments) exhibited an ANI of 93.71% (Fig. 3b). Although plasmid B of K101^T^ was only 57% the size of pSymA from *S. meliloti*, 59% (153 out of 257 sequence fragments) demonstrated an ANI of 90.80% (Fig. 3c). However, plasmid A from K101^T^ did not display appreciable orthologous sequence fragments with the pSymA or pSymB plasmids from *S. meliloti*. These data suggest that three of the four replicons from K101^T^ are related to the replicons from *S. meliloti*.

## Plasmid-carrying nodulation genes most closely related to *S. americanum*

**Fig. 4.**
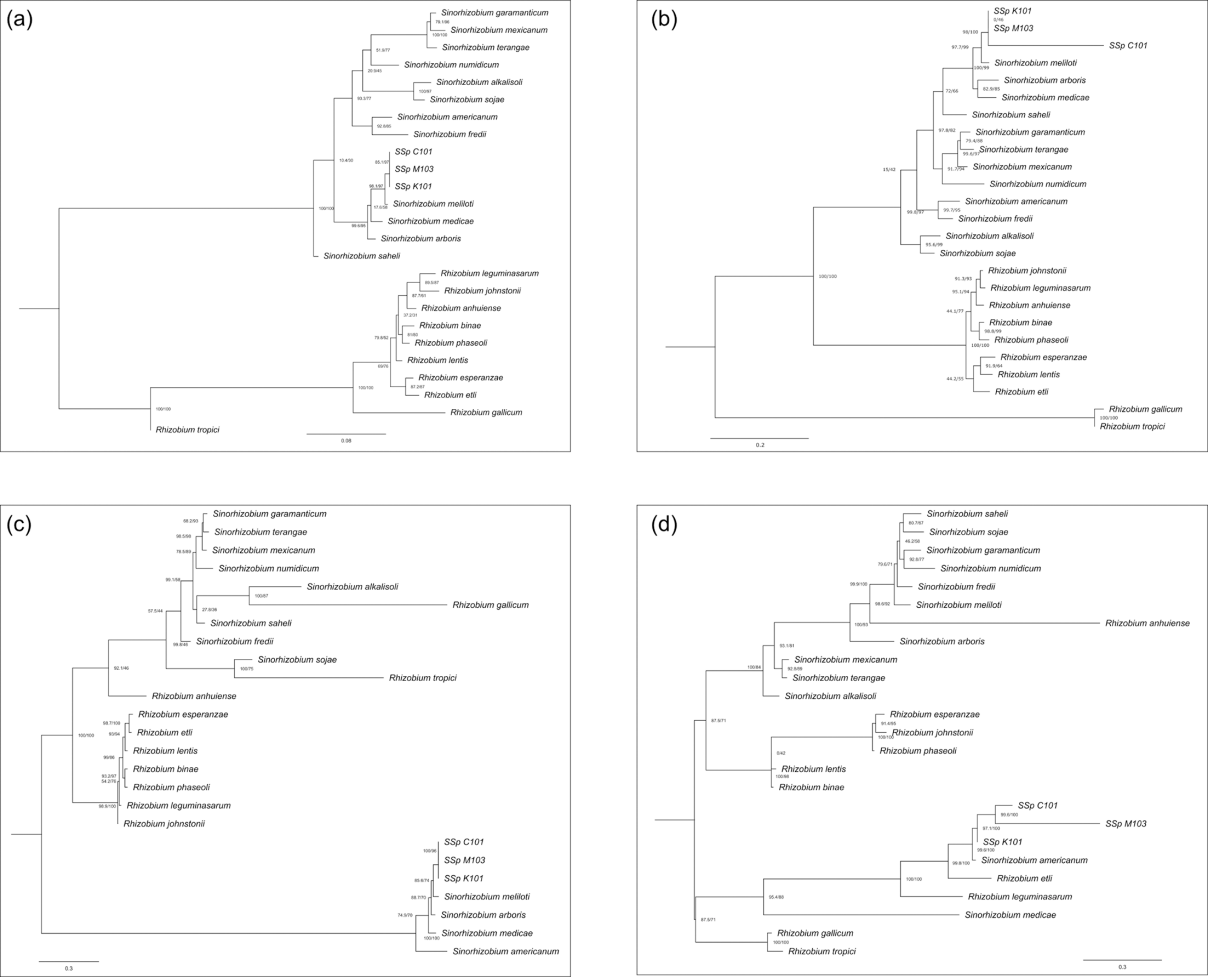
Maximum-likelihood phylogenetic tree based on the concatenation of ParAB or RepABC proteins derived from each replicon in S. prairiense C101. Percentage bootstrap values are given at branching points. (**a**) ParAB from the chromosome; (**b**) RepABC from the chromid; (**c**) RepABC from plasmid B and (**d**) RepABC from plasmid A.

To determine the relatedness of the replicons found in the *Sinorhizobium prairiense* strains, homologues of either ParAB (for the chromosome) or RepABC (for the plasmids) were identified, aligned and phylogenetically analysed to ascertain the relationships among the individual replicons. The ParAB sequences on the chromosome, the RepABC sequences on the chromid and the RepABC sequences on the larger plasmid (plasmid B) are all grouped with *S. meliloti* as their closest neighbour (Fig. 4a–c). However, the RepABC on the smaller plasmid (plasmid A) is grouped more closely with *S. americanum* (Fig. 4d).

**Fig. 5.**
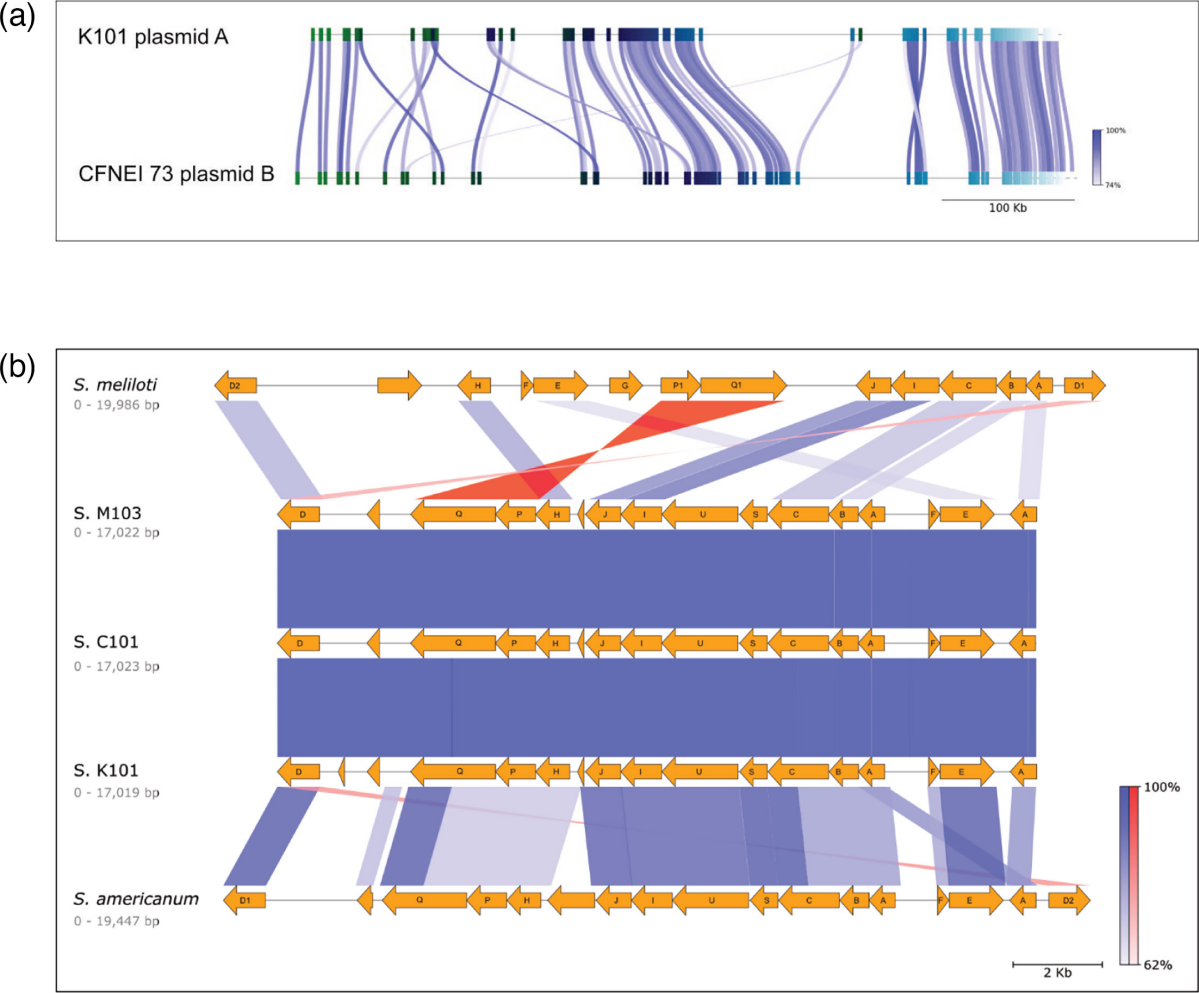
Plasmid A shares architecture with *S. americanum* CFNEI 72 plasmid B. (**a**) ANI comparison between S. prairiense K101^T^ and *S. americanum* CFNEI 72 plasmid B. (**b**) MUMmer alignment based on protein-coding sequences of the Nod regions in *S. meliloti*, *S. prairiense* K101^T^, *S. prairiense* C101, *S. prairiense* M103 and *S. americanum*. Letters represent the *nod* gene designation. Sequence similarity is shown by the saturation scale of the lines. Blue indicates similarity in the same orientation, and red indicates similarity in the opposite direction.

The *nodC* genes are traditionally used as the standard for assigning sv. in rhizobia [28]. A phylogeny consisting of *nodC* places K101^T^ in close association with sv. mediterranensis (Fig. 2a). The phylogeny of the concatenated *nodABC* provided a similar classification of the isolates, with stronger node support numbers (Fig. 2b). Since the *NodABC* genes from K101^T^ clustered most closely with *the S. americanum* strain CFNEI 73 (Fig. 2b), which is found on the plasmid most related to *S. americanum*, a FastANI comparison of plasmid A from K101^T^ with plasmid B from CFNEI 73 was carried out. It was found that 37.4% (70 out of 187 sequence fragments) showed an ANI of 85.8% (Fig. 5a). Moreover, when these regions were compared to show their relatedness with *S. americanum*’s *nod* regions, it appears that both the identity and synteny resemble those found in * S. americanum* rather than the layout and content of *S. meliloti* (Fig. 5b).

## Phenotypic characterization

The phenotypic characteristics of the isolate strains were tested and compared with those of their closest relative, *S. meliloti*. The characteristics examined included carbon source utilization, salt tolerance, antibiotic resistance, fatty acid composition and nitrogen fixation potential.

Growth profiles on different carbon sources for K101^T^, C101 and M103 were assessed using a BIOLOG plate PM01 (following the manufacturer’s recommended procedure), and the assay was performed on a BioTek EPOC2 plate reader. *S. meliloti* Rm1021 was used as an internal standard, as it is routinely used in our work [29]. When examining growth, the isolates appeared deficient in growth on dulcitol (galactitol), thymidine and uridine (Table 3). The genetic determinants for growth on dulcitol are known [30,32]. Upon further examination, it was found that the genetic components required for dulcitol metabolism were present, and a secondary test using a defined medium with dulcitol as the sole carbon source showed that K101^T^, C101 and M103 were all capable of growth with this carbon source. An interesting point of note, based on the B plate results, is that unlike *S. meliloti* Rm1021, the isolates could not grow on d-malate. We note that this was not verified on agar plates.

**Table 3.** Carbon phenotypes of *S. prairiense*

Carbon source	C101	K101^T^	M103
1,2-Propanediol	−	−	−
2-Aminoethanol	−	−	−
Glucuronamide	−	−	−
Tyramine	−	−	−
Phenylethylamine	−	−	−
l-Aspartic acid	−	−	−
l-Proline	+	+	+
d-Alanine	−	−	−
d-Serine	−	−	−
l-Glutamic acid	+	+	+
l-Asparagine	−	−	−
d-Aspartic acid	−	−	−
l-Glutamine	+	+	+
Gly-Asp	−	−	−
d-Threonine	−	−	−
Gly-Glu	−	−	−
l-Serine	−	−	−
l-Threonine	−	−	−
l-Alanine	+	+	+
Ala-Gly	−	−	−
Gly-Pro	−	−	−
l-Arabinose	+	+	+
*N*-Acetyl-d-glucosamine	+	+	+
d-Galactose	+	+	+
d-Trehalose	+	+	+
d-Mannose	+	+	+
Dulcitol	+∗	+∗	+∗
d-Sorbitol	+	+	+
Glycerol	+	+	+
l-Fucose	+	+	+
dl-α-Glycerol phosphate	−	−	−
d-Xylose	+	+	+
d-Mannitol	+	+	+
d-Glucose-6-phosphate	−	−	−
d-Ribose	+	+	+
l-Rhamnose	+	+	+
d-Fructose	+	+	+
α-d-Glucose	+	+	+
Maltose	+	+	+
d-Melibiose	+	+	+
Thymidine	−	−	−
α-Methyl-d-galactoside	+	+	+
α-d-Lactose	+	+	+
Lactulose	+	+	+
Sucrose	+	+	+
Uridine	−	−	−
d-Glucose-1-phosphate	−	−	−
d-Fructose-6-phosphate	−	−	−
β-Methyl-d-glucoside	+	+	+
Adonitol	+	+	+
Maltotriose	+	+	+
2′-Deoxyadenosine	−	−	−
Adenosine	−	−	−
*m*-Inositol	+	+	+
d-Cellobiose	+	+	+
Inosine	−	−	−
*N*-Acetyl-d-mannosamine	−	−	−
d-Psicose	+	+	+
l-Lyxose	−	−	−
d-Saccharic acid	−	−	−
Succinic acid	+	+	+
d-Glucuronic acid	−	−	−
d-Gluconic acid	−	−	−
l-Lactic acid	+	−	+
Formic acid	−	−	−
d-Galactonic acid-g-lactone	+	+	+
dl-Malic acid	+	+	+
Acetic acid	+	−	+
d-Glucosaminic acid	+	+	+
α-Ketoglutaric acid	−	−	−
α-Ketobutyric acid	−	−	−
*m*-Tartaric acid	−	−	−
α-Hydroxyglutaric acid-g-lactone	−	−	−
α-Hydroxybutyric acid	−	−	−
Citric acid	−	−	−
Fumaric acid	+	+	+
Bromosuccinic acid	+	+	+
Propionic acid	−	−	−
Mucic acid	−	−	−
Glycolic acid	−	−	−
Glyoxylic acid	−	−	−
Tricarballylic acid	−	−	−
Acetoacetic acid	−	−	−
Mono-methylsuccinate	−	−	−
d-Malic acid	−	−	−
l-Malic acid	+	+	+
*p*-Hydroxyphenyl acetic acid	−	−	−
*m*-Hydroxyphenyl acetic acid	−	−	−
Pyruvic acid	+	+	+
l-Galactonic acid-g-lactone	−	−	−
d-Galacturonic acid	−	−	−
Methyl pyruvate	+	+	+
Tween 20	−	−	−
Tween 40	−	−	−
Tween 80	−	−	−
Negative control	−	−	−

Results are presented as: (+), growth; (−), no growth.

*Utilization was negative on the PM01 plates but positive on defined agar medium containing this as the sole carbon source.

Ala-Gly, Alanine-Glycine dipeptide; Gly-Asp, Glycine-Aspartate dipeptide; Gly-Glu, Glycine-Glutamate dipeptide; Gly-Pro, Glycine-Proline dipeptide.

The soil at the three isolation sites had varying characteristics. Kelburn soil, where the type strain was isolated, consists of clay with a pH of 7.3. The Melita site was predominantly loam with a pH of 7.7, while the soil in Carmen was a mixture of sandy clay loam with a pH of 5.2. At the time of collection, the conductivity of the soil was not assessed; however, based on survey data provided by Agriculture Manitoba, the conductivity of the soil at all three locations was rated between 0 and 4 mS cm^−1^, indicating low salinity. The crops grown in the soil prior to sample collection included spring wheat, canola and oats. Soybean has been cultivated at Carmen and Kelburn in the past. Many rhizobia are sensitive to sodium chloride (NaCl), whereas some *S. meliloti* strains are very salt-tolerant and can grow in the presence of 500 mM NaCl [33]. To determine their salt tolerance, strains K101^T^, C101 and M103 were streaked onto TY agar plates containing NaCl at a range of concentrations from 10 to 500 mM and incubated at 30 °C for 2 days. The results showed that while * R. etli* CFN42 could tolerate up to 50 mM NaCl, K101^T^, C101 and M103 were like *S. meliloti* Rm1021 and able to withstand 500 mM NaCl.

The MIC of several common antibiotics was determined for the isolates. The MIC was determined on TY agar plates incubated at 30 °C for 2 days, which contained a range of antibiotic concentrations. The results indicate that all isolates share a common antibiotic resistance profile, except for K101^T^, which shows slightly greater resistance to streptomycin. The isolates exhibit significantly higher resistance to chloramphenicol, gentamicin and ampicillin compared with *S. meliloti* Rm1021 (Table 4).

**Table 4.** Resistance of *S. prairiense* to common antibiotics

	MIC (μg ml^−1^)								
**Strain**	**Sm**	**Nm**	**Km**	**Am**	**Tc**	**Cm**	**Gm**	**Sp**	**Rf**
*S. prairiense* C101	6.25	25	12.5	200	3.12	200	100	3.12	2 5
*S. prairiense* K101^T^	12.5	25	12.5	200	3.12	200	100	3.12	25
*S. prairiense* M103	6.25	25	12.5	200	3.12	200	100	3.12	25

Strains were spotted onto a series of TY plates with twofold decreasing antibiotic concentrations starting from 800 to 1.56 μg ml−1. The MIC is defined as the lowest concentration that inhibits growth. Am, ampicillin; Cm, chloramphenicol; Gm, gentamicin; Km, kanamycin; Nm, neomycin; Rf, rifampicin; Sm, streptomycin; Sp, spectinomycin; Tc, tetracycline.

To assess fatty acid composition of the isolates, liquid cultures of bacteria were grown in TY broth for 3 days at 30 °C. They were then pelleted, the supernatant was removed, and the sample was dried at 60 °C for 24 h. Approximately 40 mg of dried bacteria was used for lipid extraction. Lipids were extracted as previously described using a chloroform:methanol methylation method [34]. The extracted lipids were then analysed by gas chromatography on a Bruker 450-GC. Data are represented as the average area under the curve from biological triplicate samples. The fatty acid profile of the isolates very consistent, with the largest proportion of fatty acid types being C_16 : 0_, C_18 : 0_, C_18 : 1_ and C_18:1n7c_: palmitic, stearic, oleic and *cis*-vaccenic acid, respectively (Table S3).

The nitrogen fixation capability of the isolates was tested on common bean and alfalfa. Bean seeds were first surface-sterilized with 1% bleach and then germinated on water agar plates for 2 days. Two seedlings were transferred to autoclaved Leonard jars containing a mixture of 1 : 1 sand and vermiculite, soaked with nitrogen-free Jensen’s medium as previously described [35]. After 2 days of growth, seedlings were inoculated with ~10^7^ bacteria resuspended in 10 ml of sterile saline (0.85% w/vol). Following 1 week of growth, a plant from each pot was culled to ensure enough space for the remaining plant to grow. At 28 days post-inoculation, plants were harvested and dried at 60 °C for 48 h. The results show that K101^T^, C101 and M103 were capable of symbiotic nitrogen fixation on dry beans but not on alfalfa (Fig. S3). Additionally, dry weight accumulation on dry bean was similar to that of plants supplied with 5 mM ammonium nitrate (NH_4_NO_3_,), rather than those receiving a bacterial inoculant (Fig. S3).

## Description of *Sinorhizobium prairiense* sp. nov.

*Sinorhizobium prairiense* (prai.ri.en’se. N.L. neut. adj. *prairiense*, belonging to or originating from the Prairies, the geographic location from which the bacterium was isolated).

*S. prairiense* is a Gram-negative, non-sporulating rod. Colony morphology observed on TY medium is opaque, cream-coloured and highly mucoid after 2–3 days of growth at 28 °C. Growth occurs at NaCl concentrations of 0–3% (w/v). The strains can grow on TY and Luria Bertani (LB) media and metabolize standard carbon sources such as d-ribose, d-fructose, d-sucrose and d-xylose. The isolates are unable to grow on d-malate; K101^T^ is also unable to grow on l-lactic acid and acetic acid. The majority of the fatty acids (>90%) are found as C_16 : 0_, C_18 : 0_, C_18 : 1_ and C_18:1n7c_. The type strain of *S. prairiense,* strain K101^T^ (DSM 118657^T^=LMG 33767^T^), was isolated from the root nodules of *P. vulgaris* grown in soil from Kelburn, Manitoba, Canada. The G+C content is 62.1%, and the genome size is ~6.55 Mb.

## Supplementary material

10.1099/ijsem.0.006947 Uncited Supplementary Material 1.

## Abbreviations

ANI: average nucleotide identity
dDDH: digital DNA–DNA hybridization
sv.: symbiovar
TYGS: Type Strain Genome Server
