# Supplementary material for: Sinorhizobium prairiense sp. nov., a nitrogen-fixing symbiont of Phaseolus vulgaris isolated from Canadian prairie soil
Source: Int J Syst Evol Microbiol. 2025 Oct 30;75(10):006947. doi: 10.1099/ijsem.0.006947 (PMC12574840; doi:10.1099/ijsem.0.006947)
Supplement: Uncited Supplementary Material 1. [file ijsem-75-06947-s001.pdf]

**Supplementary Tables and Figures**

**Table S1. Copy number of symbiosis genes present in *S. sp* K101, *S. sp* C101, and *S. sp* M103.** Numbers represent copies present in the genome.

| ***nod* genes** | **C101** | **K101** | **M103** | | | ***fix/nif* genes** | **C101** | **K101** | **M103** | |
|---|---|---|---|---|---|---|---|---|---|---|
| | | | | | | | | | | |
| *nodA* | 2 | 2 | 2 | Plasmid A | | *fixA* | 3 | 3 | 3 | Plasmid A |
| *nodB* | 1 | 1 | 1 | | | *fixB* | 3 | 3 | 3 | |
| *nodC* | 1 | 1 | 1 | | | *fixC* | 1 | 1 | 1 | |
| *nodD1* | 1 | 1 | 1 | | | *fixG* | 1 | 1 | 1 | |
| *nodD2* | 1 | 1 | 1 | | | *fixH* | 1 | 1 | 1 | |
| *nodE* | 1 | 1 | 1 | | | *fixI1* | 1 | 1 | 1 | |
| *nodF* | 1 | 1 | 1 | | | *fixJ* | 1 | 1 | 1 | |
| *nodG* | 1 | 1 | 1 | | | *fixK* | 1 | 1 | 1 | |
| *nodH* | 1 | 1 | 1 | | | *fixL* | 1 | 1 | 1 | |
| *nodI* | 1 | 1 | 1 | | | *fixN* | 1 | 1 | 1 | |
| *nodJ* | 1 | 1 | 1 | | | *fixO* | 1 | 1 | 1 | |
| *nodM* | 1 | 1 | 1 | | | *fixP* | 1 | 1 | 1 | |
| *nodP1* | 1 | 1 | 1 | | | *fixQ* | 1 | 1 | 1 | |
| *nodQ1* | 1 | 1 | 1 | | | *fixT* | 1 | 1 | 1 | |
| *NodS* | 1 | 1 | 1 | | | *fixU* | 1 | 1 | 1 | |
| *NodU* | 1 | 1 | 1 | | | *fixX* | 1 | 1 | 1 | |
| *nfeD* | 1 | 1 | 1 | Chromid | | *SMa1142* | 1 | 1 | 1 | |
| *nodP2* | 1 | 1 | 1 | | | *nifA* | 1 | 1 | 1 | |
| *nodQ2* | 1 | 1 | 1 | | | *nifB* | 1 | 1 | 1 | |
| *nsrA* | 1 | 1 | 1 | | | *nifD* | 1 | 1 | 1 | |
| *SMb20825* | 1 | 1 | 1 | | | *nifE* | 1 | 1 | 1 | |
| *SMb21110* | 1 | 1 | 1 | | | *nifH* | 1 | 1 | 1 | |
| *aqpZ1* | 1 | 1 | 1 | Chromosome | | *nifK* | 1 | 1 | 1 | |
| *glmS* | 1 | 1 | 1 | | | *nifN* | 1 | 1 | 1 | |
| *nodN2* | 1 | 1 | 1 | | | *nifX* | 1 | 1 | 1 | |
| *nolR* | 1 | 1 | 1 | | | *nifS* | 1 | 1 | 1 | Chromosome |
| *ssrA sra* | 1 | 1 | 1 | | | | | | | |

## Table S2. Accession numbers used for Figure 2.

| | Genome Accession RefSeq | *nodC* locus tag | NodC protein | NodB protein | NodA protein |
|---|---|---|---|---|---|
| *S. saheli* LMG 783 | GCF_001651875.1 | ATB98_RS22545 | WP_084435703.1 | WP_066877694.1 | WP_066877698.1 |
| *S. alkalisoli* YIC4027 | GCF_008932245.1 | EKH55_RS27140 | WP_069457971.1 | WP_069457972.1 | WP_069457973.1 |
| *S. sojae* CCBAU 05684 | GCF_002288525.1 | SJ05684_RS27985 | WP_014858070.1 | WP_010875356.1 | WP_015633488.1 |
| *S. glycinis* CCBAU23380 | GCF_001651865.1 | AU381_RS13585 | WP_014858070.1 | WP_010875356.1 | WP_015633488.1 |
| *S. garamanticum* LMG 24692 | GCF_029892065.1 | PZN02_RS31425 | WP_280663451.1 | WP_280663452.1 | WP_280663453.1 |
| *S. numidicum* LMG 27395 | GCF_029891955.1 | PYH38_RS29415 | WP_280736409.1 | WP_280736407.1 | WP_280736406.1 |
| *S.numidicum* CIP 109850 | GCF_029892045.1 | PYH37_RS29525 | WP_280736409.1 | WP_280736407.1 | WP_280736406.1 |
| *S. medicae* USDA 1037 | GCF_007827695.1 | FB009_1426 | WP_011970892.1 | WP_011970891.1 | WP_011970890.1 |
| *S. meliloti* Rm 1021 | GCF_000006965.1 | SM_RS27110 | WP_010967454.1 | WP_003532851.1 | WP_010967455.1 |
| *S. meliloti* USDA 1002 | GCF_017876815.1 | JOH52_RS29145 | WP_017266288.1 | WP_014531648.1 | WP_011970890.1 |
| *S. psoraleae* CCBAU 65732 | GCF_013283645.1 | ILFOPFJJ_RS33470 | WP_173514698.1 | WP_173514697.1 | WP_173514826.1 |
| *S. arboris* LMG 14919 | GCF_000427465.1 | SINAR_RS0129120 | WP_028002360.1 | WP_028002359.1 | WP_028002358.1 |
| *S. kostiense* DSM 13372 | GCF_017874595.1 | J2Z31_RS26425 | WP_209606300.1 | WP_209606298.1 | WP_209606295.1 |
| *S. terangae* CB 3126 | GCF_029714365.1 | QA637_RS30535 | WP_346283797.1 | WP_283067648.1 | WP_283067650.1 |
| *S. chiapasense* ITTG S70 | GCF_036488675.1 | RB548_RS24655 | WP_331375576.1 | WP_331375577.1 | WP_331375578.1 |
| *S. mexicanum* ITTG R7 | GCF_013488225.1 | FKV68_RS23885 | WP_180942081.1 | WP_180942082.1 | WP_180942083.1 |
| *S. americanum* CFNEI156 | GCF_001651855.1 | ATC00_RS22405 | WP_064254499.1 | WP_064254500.1 | WP_037390235.1 |
| *S. americanum* CFNEI 73 | GCF_001889105.1 | SAMCFNEI73_RS19310 | WP_064254499.1 | WP_064254500.1 | WP_037390235.1 |
| *S. meliloti* 4H41 | GCF_000375585.1 | B075_RS0120750 | WP_018097533.1 | WP_018097534.1 | WP_018097535.1 |
| *S. fredii* GR64 | GCF_000219415.3 | SFGR64A_RS31875 | WP_028003553.1 | WP_018097534.1 | WP_018097535.1 |
| *S. meliloti* GVPV12 | GCF_000428005.1 | A3C9_RS0115305 | WP_028003553.1 | WP_018097534.1 | WP_018097535.1 |

**Table S3. Fatty acid composition of *S. prairiense*.**

| | C101 | K101 | M103 |
|---|---|---|---|
| C10:0 | -[a] | - | - |
| C12:0 | - | - | - |
| C14:0 | 0.1 (0.1) | 0.1 (0.1) | 0.1 (0.1) |
| C14:1 | - | - | - |
| C15:0 | - | 0.1 (0.0) | - |
| **C16:0** | **15.9 (1.1)**[b] | **13.1 (1.9)** | **11.8 (0.4)** |
| C16:1 | 0.33 (0.2) | 0.3 (0.0) | 0.5 (0.2) |
| C16:1t | 0.1 (0.0) | 0.1 (0.0) | 0.1 (0.0) |
| C17:0 | 0.4 (0.0) | 0.4 (0.0) | 0.4 (0.0) |
| **C18:0** | **20.7 (5.0)** | **17.2 (3.0)** | **13.8 (1.0)** |
| **C18:1** | **2.5 (0.9)** | **1.5 (0.5)** | **1.7 (0.7)** |
| **C18:1n7c** | **57.9 (6.3)** | **66.0 (4.5)** | **70.6 (0.6)** |
| C18:2 | 0.6 (0.2) | 0.4 (0.3) | 0.3 (0.1) |
| C20:0 | 0.2 (0.1) | 0.1 (0.1) | 0.2(0.1) |
| C20:1 | - | - | - |
| C20:4 | - | - | - |
| C20:5 | 1.0 (0.1) | 0.6 (0.2) | 0.5 (0.1) |

Values represent the % of the total sample area under the curve per sample with the standard deviation in brackets.

[a] -, not detected, or less than 0.04% of the total sample

[b] Values in bold represent the highest proportion of the sample, accounting for at least 97% of the fatty acids detected. Values may not sum to 100% due to rounding off of values.

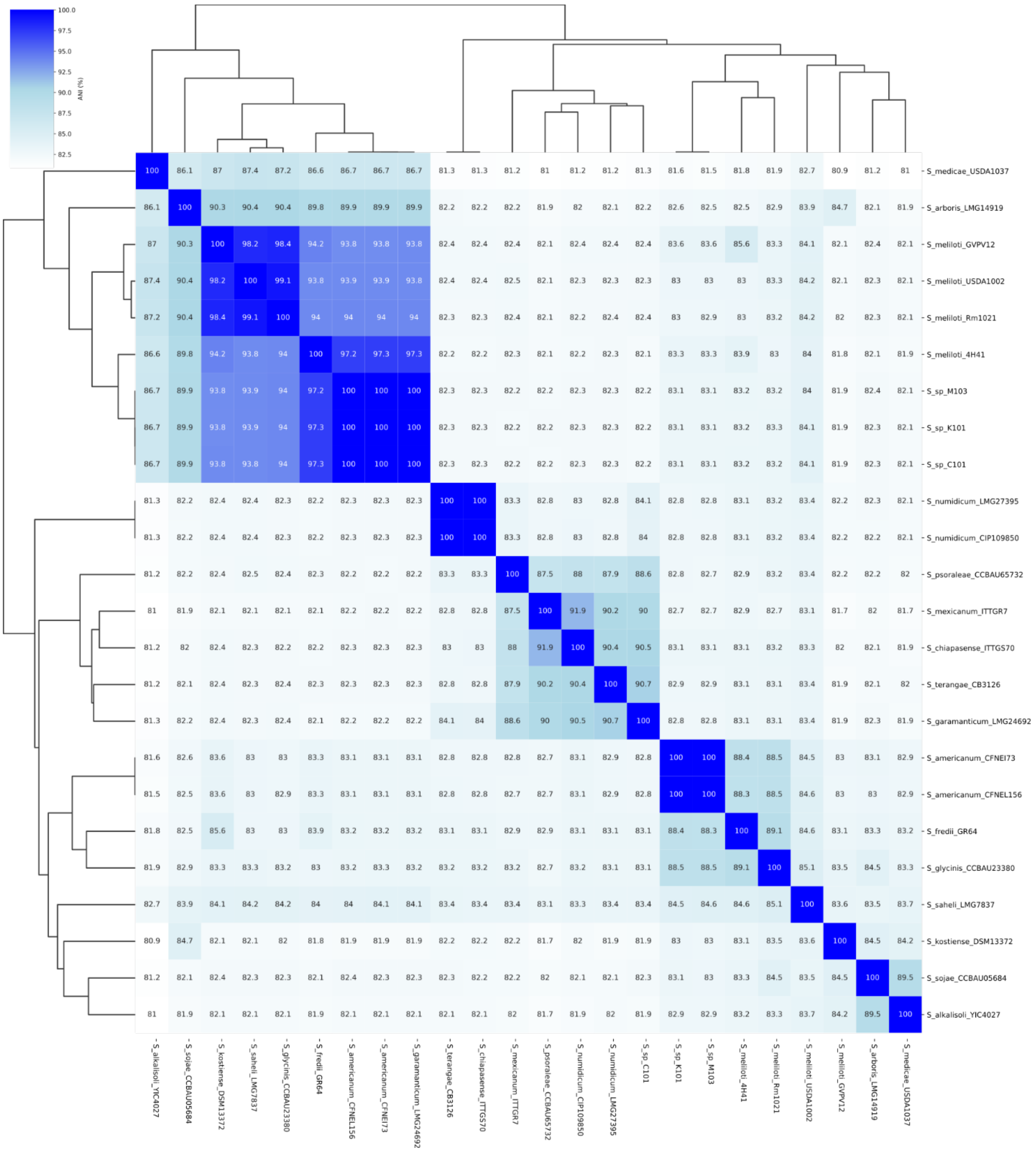


**Figure S1.** Heat map of whole genome average nucleotide identity (made using ANIclustermap [1] and fastANI) comparing several *Sinorhizobium* strains and the isolates designated as S_sp_C101, S._sp_K101, and S_sp_M103.

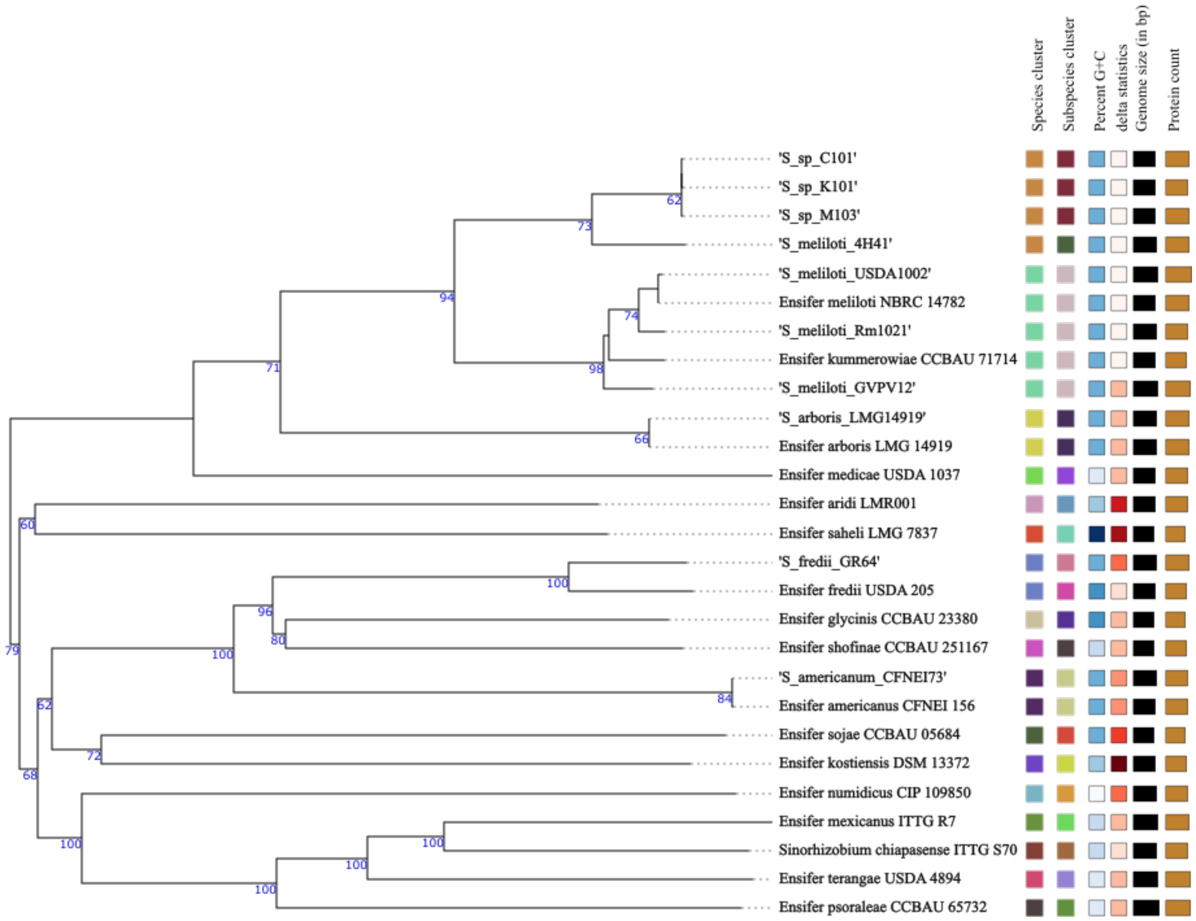


**Figure S2. TYGS whole genomes phylogenetic tree.** Tree inferred with FastME 2.1.6.1 [2] from Genome BLAST Distance Phylogeny (GBDP) distances calculated from genome sequences. The branch lengths are scaled in terms of GBDP distance formula d5. The numbers above branches are GBDP pseudo-bootstrap support values > 60 % from 100 replications, with an average branch support of 77.3 %. The tree was rooted at the midpoint [3]. Strains with apostrophes were submitted to the server, all other strains were selected by the server for comparison.

A.

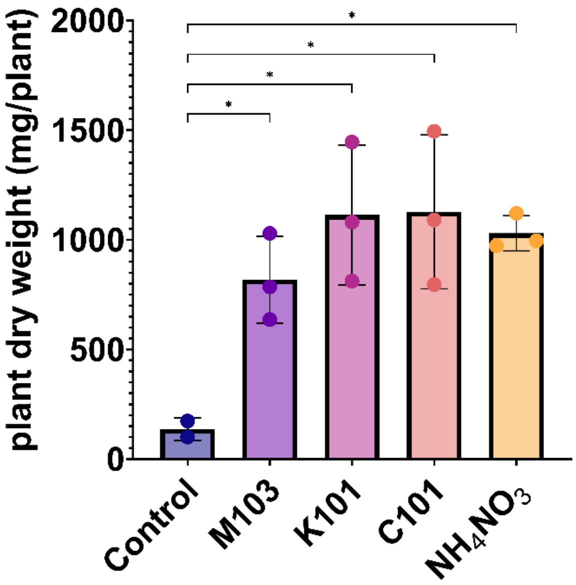


B.

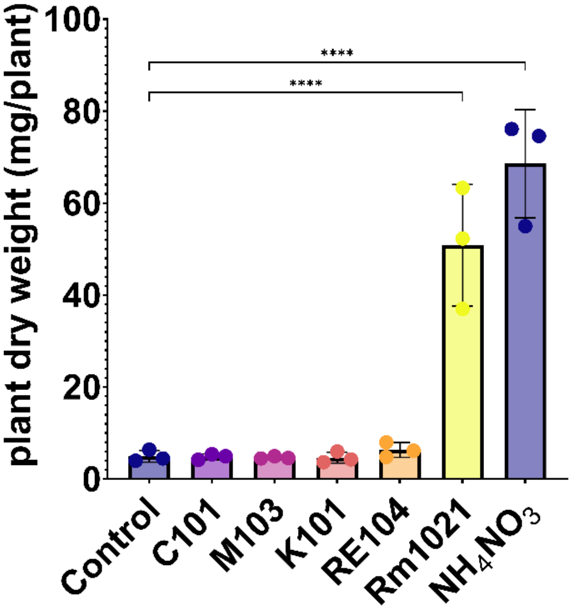


**Figure S3.** Nitrogen fixation capacity as measured by plant dry weight after 28 days. Strains used are listed on the x-axis. Control, plants grown in nitrogen-deficient conditions; $NH_4NO_3$, Uninoculated plants supplemented with 5 mM ammonium nitrate at the start of the experiment. An ANOVA with a post hoc Dunnett test was used to measure significance A) Dry bean * $p < 0.01$; B) Alfalfa **** $p < 0.0001$.

**Supplemental Literature Cited**

[1] Shimoyama, Y. (2022). ANIclustermap: A tool for drawing ANI clustermap between all-vs-all microbial genomes. https://github.com/moshi4/ANIclustermap

[2] Lefort V, Desper R, Gascuel O. FastME 2.0: A comprehensive, accurate, and fast distance-based phylogeny inference program. Mol Biol Evol. 2015;32: 2798–2800. DOI: 10.1093/molbev/msv150

[3] Farris JS. Estimating phylogenetic trees from distance matrices. Am Nat. 1972;106: 645–667.
